# Perceptions of cervical screening uptake amongst South Asian women in Ontario, Canada: a concept mapping study

**DOI:** 10.1186/s12889-025-21448-6

**Published:** 2025-01-22

**Authors:** Kimberly Devotta, Aisha Lofters, Jacqueline Bender, Patricia O’Campo

**Affiliations:** 1 University of Toronto, 155 College St Room 500, Toronto, ON M5T 3M7 Canada; 2 Women’s College Hospital, 76 Grenville St, Toronto, ON M5S 1B2 Canada; 3 University of Toronto, 500 University Ave, Toronto, ON M5G 1V7 Canada; 4 University Health Network, 190 Elizabeth St, Toronto, ON M5G 2C4 Canada; 5 St. Michael’s Hospital, Unity Health Toronto, 30 Bond Street, Toronto, ON M5B1W8 Canada

**Keywords:** Concept mapping, Cervical screening, Health equity, Women’s health, Community engagement, South Asian women, Participant-driven

## Abstract

**Background:**

Regular cervical screening can significantly reduce the onset and prevalence of cervical cancer. In Ontario, Canada, South Asian women have the lowest rates of cervical cancer screening among major ethnic groups in the province.

**Methods:**

Using an innovative and participant-driven method called Concept Mapping (CM), we set out to understand how the lives and experiences of South Asian women living in Ontario shape their decisions around getting screened for cervical cancer. We engaged over 70 South Asian women and people who serve them in healthcare and community, to drive the CM process.

**Results:**

Participants brainstormed 45 unique and distinct statements. Through sorting and map interpretation, participants identified and interpreted 6 clusters amongst the statements: (1) Personal beliefs and misconceptions around cervical cancer; (2) Education and knowledge issues around cervical cancer; (3) Cultural beliefs and influences specific to sexual health; (4) Barriers to prioritizing uptake of cervical screening; (5) System/ infrastructure gaps or inadequacies; and (6) Lack of comfort and supportive relationships in healthcare. Additional analysis shows us the interrelationships between the ideas. Statements within the clusters about education and knowledge issues around cervical cancer, personal beliefs and misconceptions, as well as cultural beliefs and influences specific to sexual health are viewed as distinct beliefs with clear effects on the uptake of cervical screening. More complex interrelationships are seen with the cluster of statements about barriers to prioritizing uptake of cervical screening.

**Conclusions:**

As Ontario and many other jurisdictions around the world seek to strengthen cervical screening efforts in line with national and international goals to eliminate cervical cancer by 2040, it is critical to address underscreening. This CM study recognizes the value of engaging those most impacted by an issue, to identify and prioritize how and where to intervene to address low rates of cervical screening. To address underscreening we need to design multi-level interventions that address the identified ideas and the interrelationships among them.

## Background

With a lengthy pre-cancerous stage, regular screening can dramatically reduce both incidence and prevalence of cervical cancer. In 2024, approximately 1600 women will be diagnosed with cervical cancer in Canada, and an estimated 400 women will die from it [1]. Today, cervical has become the fastest increasing cancer in females and we know that cervical cancer diagnoses and deaths are avoidable through regular screening [2]. In the province of Ontario, Canada, the Ontario Cervical Screening Program (OCSP) was established in 2000 and at the time, 59% of eligible women were getting screened for cervical cancer in the province [3]. Participation rates, however, peaked at 67% in 2007–2009, and have remained stable at approximately 60% since 2013 - well below provincial targets of 80 to 85% [3]. Many groups, including newcomers, people who are older, of lower socio-economic status, in poorer health and otherwise marginalized, are amongst the most under- or never-screened (UNS) for cervical cancer in Ontario [4]. Cervical cancer is one of the few cancers with a readily detectable and treatable precursor stage, making prevention and screening the most reliable strategy [5]. The purpose of screening is to reduce the risk of cervical cancer by looking for, and treating, lesions that have the potential to become cancerous. The OCSP and other organized screening programs throughout Canada, use cytology testing (e.g. Pap tests) and this has been largely responsible for a dramatic decline in cervical cancer incidence as pre-cancerous cells can be detected and treated early [6]. In 1972 age-standardized cervical cancer incidence were 22.3 per 100,000 and in 2006 were 9.4 per 100,000, marking a 58% decline [7]. After seeing a 30-year decline, the incidence of cervical cancer has been increasing around 3.7% per year, since 2015, becoming the fastest increasing cancer for females [2]. This change has been attributed to multifactorial drivers including suboptimal screening uptake and lack of follow up after screening [2].

At the start of this study (September 2019), the OCSP was recommending that everyone with a cervix who has been sexually active, commence cytology-based screening (i.e. a Pap test) at the age of 21 (they currently recommend commencing screening at the age of 25). In Ontario, Pap tests are typically done by an individual’s primary care provider – family doctor or nurse practitioner – and sometimes in public health units, sexual health clinics and community health centres in Ontario. In some rural northern parts of the province, Pap tests are offered in mobile screening coaches [1]. Pap tests are publicly funded through the Ontario Health Insurance Plan (OHIP) [8].

Using data from the 2017 Canadian Community Health Survey, Benjamin et al. [9] found that women identifying as South Asian were the least likely to self-report being adherent to cervical screening recommendations in Ontario (OR: 0.44, 95%CI: 0.20–0.94). South Asian women have the lowest rates of cervical cancer screening among major ethnic groups in Ontario, and a higher burden of cervical cancer [10–14]. In their study of cancer screening in Ontario, Lofters et al. [13], found that South Asian patients were the most vulnerable to underscreening for cervical cancer, as the adjusted odds ratio of screening for South Asian women compared to non-immigrant women in urban primary care practices in Ontario, was 0.61 (95% CI 0.59–0.64).

Low rates of cervical screening have been attributed to different factors within people’s lives and experiences. In their studies of South Asian women living in Hong Kong, Chan and So [15, 16] identified a widely held and impactful perception amongst South Asian women that screening is unnecessary if one has no symptoms. In their multimedia intervention study of new and repeated cervical screening participation amongst South Asian women, Chan and So [15] found that participants believed that screening was unnecessary because their health status was fine and they were not experiencing any physical symptoms or discomfort. Additionally, in their cross-sectional study where they surveyed close to 800 South Asian women, Chan and So [16] found the belief they did not need a test if they felt well, to be one of the most significant barriers associated with a participant’s uptake of cervical screening.

Additionally, emotion-laden responses such as fear, anxiety and shyness around getting a Pap test have been found to prevent many South Asian women from being screened [5, 10, 17–21]. In a mixed-methods study around underscreening in Ontario, South Asian women, amongst other under- or never-screened women, cited reasons such as privacy and discomfort with pelvic examinations as reasons for avoiding cervical screening [22, 23]. Relatedly, studies of other groups of women have also shown how superstitious beliefs can prevent screening. In their study of Iranian women’s perception of cervical cancer prevention, Khazaee-Pool and colleagues [24] found that women believed discussing cervical cancer could put them at risk of developing it, or that the ‘evil eye’ could cause cervical cancer. While this study does not include South Asian women, it is possible that there may some similar beliefs due to similarities in culture.

Among South Asian immigrants to Canada, the USA and the UK, acculturation – as measured by an individual’s length of residence and language mastery – has been studied as a determinant of screening uptake, with greater acculturation leading to greater uptake [20, 25–28]. Other factors associated include the impact of personal experiences with ill health and previous screening, leading to a greater risk perception and conviction around the benefits of screening [29]. Feelings of self-efficacy to do something positive for one’s own health can be empowering and also encourage women to seek out screening [30]. Lastly, challenges with the Pap test itself can include shyness, physical discomfort and feeling like their privacy has been invaded, leading many to avoid cervical screening [22, 23]. These studies highlight many reasons why people may not be screened and are often focused specifically on the Pap test itself and not some of the contextual factors that shape screening decisions. Additionally, a closer look at Ontario is needed to uncover more unique and tailored approaches to addressing underscreening. While many qualitative, quantitative and mixed methods studies have identified themes in underscreening and barriers to Pap tests, what is needed is an approach that better engages South Asian women and other relevant stakeholders to comprehensively identify the range of direct and indirect factors on cervical screening uptake to better understand the how screening decisions are made within the context of people’s lives.

The persistence of these rates of underscreening make it ever more important to understand how the different facets of South Asian women’s lives and experiences impact their cervical screening uptake. To effectively address the complexities that cause these disproportionate rates, it is necessary to understand South Asian women’s own perspectives on screening to improve organized screening and inform effective interventions in the province and internationally.

Using concept mapping (CM), we aimed to engage a range of stakeholders to build a conceptual framework that reflects how South Asian women’s lives and experiences impact their cervical screening practices in Ontario, and to better understand the interrelationship of these different ideas. We need to move beyond traditional methods that simply identify and quantify reasons for underscreening, to use methods that allow people to lead, collaborate and develop action plans to address the issues they are experiencing and uncover the interrelationships amongst known and newly identified factors. This is the first concept mapping study to focus on the decision-making of South Asian women in Ontario around cervical screening, to understand the larger picture of how aspects in their lives and experiences impact cervical screening.

In this study, South Asian identity refers to those that self-identify with a South Asian ancestry (i.e., not necessarily of South Asian birth). South Asian ancestry can include the following countries: Afghanistan, Bangladesh, Bhutan, India, Maldives, Nepal, Pakistan, Sri Lanka. This also includes people who can trace their origins back to South Asian countries, such as Indo-Guyanese people. South Asian refers to ancestry and includes those that have immigrated to Canada and those that were born in Canada.

This CM study is guided by the research question: how do the lives and experiences of South Asian women living in Ontario shape their decisions around getting screened for cervical cancer? Additionally, we set out to understand how these experiences cluster into larger themes.

## Methods

### Study design

The social-ecological model (SEM) posits that there are multiple levels of influence on health behaviours [31]. This is particularly important when understanding uptake of cervical screening amongst South Asian women, as multiple influences at multiple levels of their social and physical environments can encourage or create barriers to cervical screening. As such, the use of SEM is reflected in how we explored the multi-level aspects of the lives and experiences of South Asian women that impact uptake of cervical screening.

**Fig. 1:**
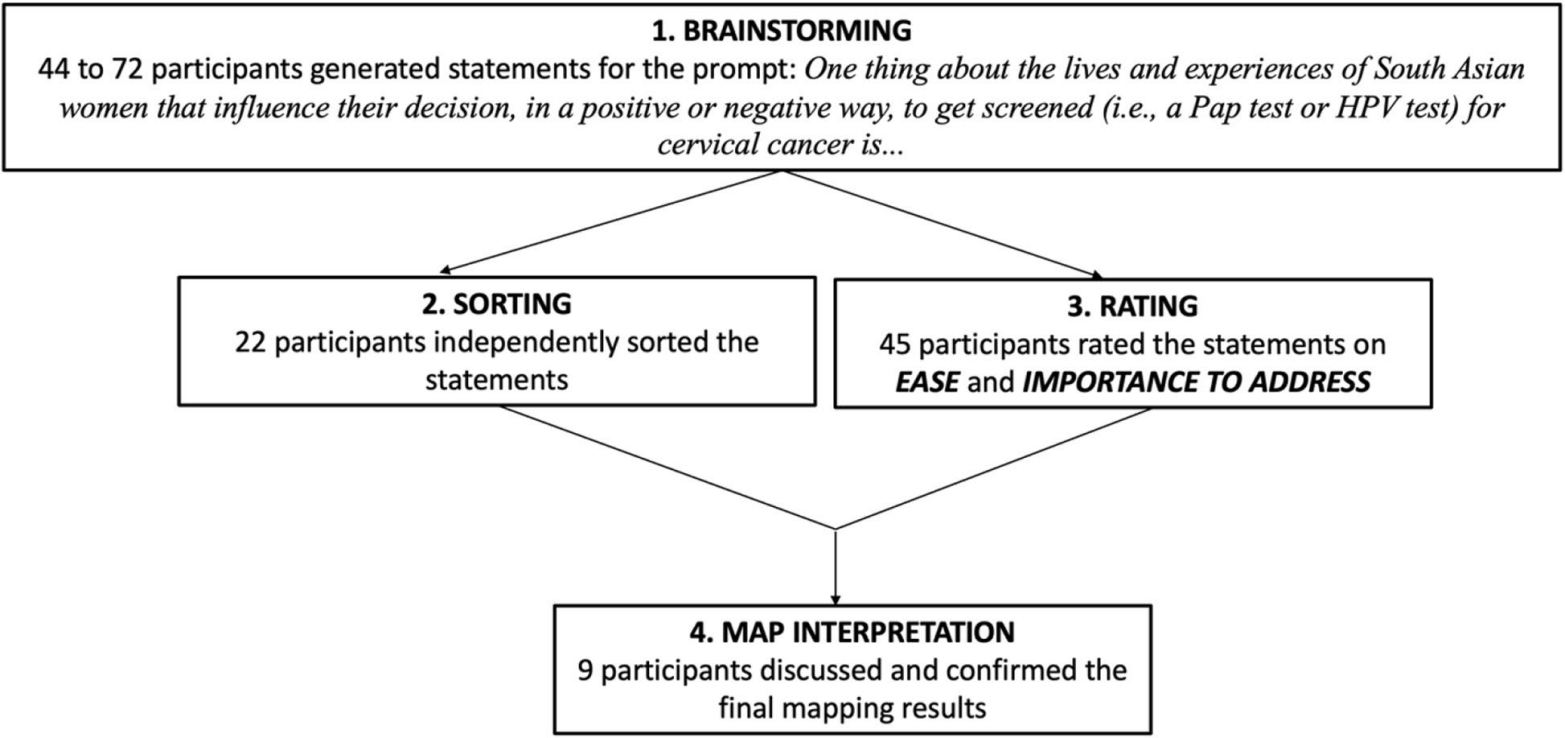
Flowchart of order of concept mapping activities

For this study, a Concept mapping (CM) study design was used. CM was developed by Trochim and Kane [32, 33] to collect ideas from a group of individuals, identify how they organize the interrelationship amongst the ideas, and to represent their group thinking with graphics. The result of this method is a conceptual framework that reflects how a group views a particular topic [33]. CM has many participatory aspects. Participants are involved in the generation of ideas, and the organizing and prioritizing of data, labelling of findings and discussion of relevance. They are given an opportunity to challenge results and discuss application of findings. The processes within CM combine qualitative approaches with quantitative analytical tools to create a display of the relationship between ideas [32]. It provides an opportunity to develop a joint meaning and group consensus [34]. We emphasized the participatory aspects of CM in our study through engaging participants in the four main activities that are described below ("Data analysis" section, Fig. 1): brainstorming, sorting, rating and map interpretation.

CM is unique and innovative in its qualitative approach. CM is comprised of activities where participants work independently avoiding group dynamic issues of conformity bias, as well as participants needing to process their perceptions and express their personal experiences in front of people (e.g., researchers, other participants) [35]. This is particularly useful when studying cervical screening amongst South Asian women, as it can be a very difficult topic for people to discuss.

One of the major strengths of CM is the inclusion of participants in the interpretation and analyses. Unlike researchers leading coding of interview and focus group data, in CM the researcher is there to manage the process while participants contribute directly to data analysis, discussion and interpretation of the findings [35]. Additionally, CM moves beyond simply identifying and exploring themes, to also include analysis of how the themes relate to one another, and this is a stronger approach to understanding complex issues than interviews and focus groups [35].

Ethics approval for this study was granted by the Research Ethics Board at the University of Toronto (REB# 43281).

### Sampling strategy and recruitment

While initially designed for groups of 40 or fewer in-person participants, online tools make it possible to have larger and/or geographically dispersed groups [33]. For this study, a range of stakeholders were engaged in the CM process to understand how the lives and experiences of South Asian women influence their decisions around cervical screening. The stakeholders included: (i) South Asian women living in the Greater Toronto Area (GTA), (ii) community champions – trusted, female members of the South Asian community with pre-existing connections with local community groups and organizations, (iii) people who work in organizations that serve South Asian women in the GTA, and (iv) healthcare providers serving South Asian patients. Since the goal of CM is to achieve a broad sample of ideas and not a representative sampling of persons, nonrandom sampling was used [33]. Purposive sampling for heterogeneity was used to select participants that were most likely to yield appropriate and useful information [33, 36].

Eligibility criteria for South Asian women included: self-identify as South Asian, and is or has ever been eligible for cervical screening in Ontario (at least 21 years of age, has been sexually active, has a cervix). All other participants such as healthcare providers and community service providers, had to identify as being in a role that works or serves South Asian women. These participants were also asked to gauge their familiarity with cervical cancer screening amongst South Asian women. All participants had to be at least 18 years of age and speak conversational English.

### Recruitment procedures

Recruitment primarily occurred through poster distribution at groups and organizations that have a high South Asian client population, and through word-of-mouth. A community champion shared the flyer with more informal groups on social media, WhatsApp, and in her personal circle. Service providers were primarily recruited through email and poster distribution on mailing lists at hospitals, primary care teams and other health and social services.

### Demographics

Each time they participated, participants were asked to select one of the following five categories: (i) I identify as South Asian; (ii) I work in a role or an organization that serves South Asian women and I identify as South Asian; (iii) I work in a role or an organization that serves South Asian women and I do not identify as South Asian; (iv) I work as a primary care provider and I identify as South Asian; or (v) I work as a primary care provider and I do not identify as South Asian. The first category is considered ‘service users’ and the remaining categories are referred to as ‘service providers.’ Service providers were also asked how long they have been in their area of work, and service users were asked if they had ever had a Pap test. All participants were asked their age and gender identity. Due to the limitation of the Group Wisdom platform only allowing the collection of a small amount of demographics, we did not have non-South Asian participants go on to specify their ethnicity. In the sorting and map interpretation activities (described below), service providers were additionally asked for the approximate percentage of South Asian people that they serve, and further details about their roles in healthcare and the community.

### Concept mapping activities

To prepare for CM, the researcher must first determine the focus, which is typically a sentence completion prompt or a directive that asks participants for special statements or expressions of interest about a topic [32]. There are four data collection activities that follow: brainstorming, sorting, rating and map interpretation. This is summarized in Fig. 1.

All CM activities were completed between September 2022 and August 2023. Concept Systems Group Wisdom was the online platform used for participation and storage of data. The lead author (KD) checked the platform for new responses, on a daily basis. This paper presents the data from the brainstorming, sorting and map interpretation activities. The brainstorming data show us what experiences in the lives of South Asian women were identified as impacting cervical screening, and the sorting data shows us how these brainstormed ideas cluster into larger themes. Analysis of the rating data has been published elsewhere [37, 38].

#### Brainstorming activity

The objective of the brainstorming round was to encourage participants to think broadly about factors within the lives and experiences of South Asian women that impact their decision to get screened for cervical cancer. This round was done completely online, using the Group Wisdom online platform. With input from the study team and members of the community, the following prompt was developed, and participants were asked to provide up to 10 responses to the prompt: *One thing about the lives and experiences of South Asian women that influence their decision*,* in a positive or negative way*,* to get screened (i.e.*,* a Pap test or HPV test) for cervical cancer is…* The brainstorming activity used an anonymous link so participants could feel more comfortable to express their thoughts and to also make participation low-barrier (e.g. no passwords or usernames). This was particularly important for this activity, as we wanted to include as many people as we could. The demographic questions were set up as the first part of the activity, so people did not accidentally miss the questions. These were submitted and then participants were taken to the brainstorming activity. With this link, participants were able to see what statements had already been collected from other participants and could provide additional statements that they believed were relevant. Participants could also review the existing statements and choose to not add anything additional. Lastly, they could come back to the activity to add in additional statements they thought of later. The initial brainstormed statements were reduced into a shorter manageable number of statements composing the master list that was used in the remaining CM activities.

#### Sorting activity

In the sorting activity, participants were asked to provide their perceptions on the similarity between the items in the master list. The purpose of this was to identify how participants view the interrelationship of the ideas [33]. Participants were asked to sort the statements from the master list described above into piles that made sense to them. The sorting task was done independently and were given the choice to complete this via GroupWisdom or during an in-person event using paper and pencil. Most participants completed this activity online with a personalized link where they could save their work and return to it later. Due to the relatively challenging nature of this task, an in-person group was held in a community centre, so people could complete the activity with a member of the team (KD) there to facilitate and provide real-time instruction. Findings from the application of hierarchical cluster analysis enabled the generation of point cluster maps. These maps show us how participants sorted the statements and thought about them thematically. A group of participants reviewed, revised and confirmed the map in the final CM activity: map interpretation. During this session, participants also discussed and agreed on a label for each grouping (i.e., cluster).

####  Data collection

Data collection and analyses were managed through Concept Systems Group Wisdom. The group map interpretation session was held over Zoom and facilitated by the lead author (KD). Participants were given a letter of information before each activity and provided their implied consent through their participation in the activity. Participants were given an e-gift card upon completion of each activity: $30 for brainstorming, $40 for sorting and $30 for map interpretation. Participants were not required to complete all the CM activities. Some participants who were recruited during the brainstorming activity participated in all activities of the study whereas others participated in 1 to 3 activities.

Figure 1 describes the order of the concept mapping activities.

### Data analysis

#### Creation of master list from brainstormed statements

The data from the brainstorming round was cleaned to remove duplicate, incomplete or off-topic responses. The purposes of this are to obtain a list of unique items, where each item represents a single idea that is relevant to the CM focus, and to reduce the number of items to a manageable list for participants to sort and rate [27]. Two research team members (KD and AL) reviewed each statement that was removed, for agreement. A meeting with the research team was held to confirm the decisions made during item reduction. The draft master list was then reviewed for clarity by the community champion, a service provider and two members of the research team (AL and PO) to create the final master list to be used in the subsequent CM activities (e.g. sorting). Minor edits were made for clarity, including grammar.

#### Hierarchical cluster analysis of sorting data

The sorting activity is the basis for the point and cluster maps that are created in GroupWisdom. First, a similarity matrix is created that indicates the number of participants that sorted each pair of statements together [29]. Then multidimensional scaling (MDS) of the summed up similarity matrix uses the similarity data and represents them as distances in Euclidean space, locating each statement as a separate point on a two-dimensional (X, Y) map [33, 39]. In MDS the key diagnostic measure is the stress value. This is a metric for indicating the degree to which the MDS fits the original similarity matrix [40]. A lower stress value is preferred and suggests a better overall fit [33]. Kane and Trochim state that 95% of concept mapping projects are likely to have stress values that range between 0.205 and 0.365 [33]. Hierarchical cluster analysis groups individual statements on the point map into clusters of statements that reflect similar concepts [40].

The location of the statement points on the map and the resulting distance between them, show us how people sorted them and thought about their relatedness. Points that are far apart mean than people did not frequently group these together, and therefore did not see them as related. Points that are closer together were frequently sorted together, indicating that participants often found them related to each other. In this step of the analysis, the researcher is able to see the solutions for different number of groupings and make decisions about which set of groupings best fit the data, that is yields the clusters with the best internal cohesion for the statements, based off where the point representing the statement is located. This shows us how participants think about the ideas thematically.

#### Bridging and anchoring analysis

After MDS and the hierarchical cluster analysis, a bridging value ranging from 0 to 1 is computed for each statement and then an average for each cluster. This bridging and anchoring analysis describes how each statement and cluster on the map is related to the statements around it [40]. ‘Anchors’ have lower values which indicate the statement was more often sorted by participants with others close to it on the map [40]. These reflect greater internal cohesion of a cluster. Higher values known as ‘bridges’ indicate statements that were less often sorted together by participants and sorted with statements on the other side of the map [40].

#### Map interpretation session

The final activity was a session where a group of participants were brought back to see the CM results and have a discussion on its utility [33]. This session was completed over Zoom videoconferencing to allow more participants to attend. This activity is a form of member checking. During this session, participants were presented with some of the draft cluster maps that were suggested by GroupWisdom, to discuss and choose a version that makes the most sense to them. Once a map was agreed upon, participants came up with labels for each cluster and discussed regions within the map.

## Results

### Participant sample

Since the main goal of the brainstorming activity was to gather a range of ideas, we chose to use an anonymous link to make participation low barrier and more inclusive. This meant that people could participate without signing up for an account or participant ID. It also helped to reassure participants that their responses would not be tied directly to them, further encouraging them to be more open about a topic that can often be shrouded in stigma. Participants still completed a eligibility screening questionnaire before being emailed the link, so we were still able to have an idea of who we were reaching in our recruitment. During the brainstorming activity, submitting the demographics questions was one step in the process of accessing the main activity. However, for those who came back multiple times to brainstorm additional responses, they would have had to submit the demographic questions again, before accessing the activity. As a result, it is not possible to know exactly how many people participated, as coming back to the activity more than once would count the same participant multiple times in the demographic questions. We know participants came back multiple times as there were more completed demographics questions than people that were invited to participate. Using the completed demographics questions and available time stamps, the number of participants estimated for the brainstorming round is between 44 and 72: 44 sets of demographic questions were completed and an accompanying list of brainstormed statements submitted; an additional 12 sets of demographic questions were completed and at least one statement was added to the existing list but the activity was not submitted; 16 sets of demographic questions were completed but no other action was taken, which may mean the statements were looked at with nothing additional to add. We recruited 22 participants to complete the sorting activity. Half of them were service providers and the other half were service users. The map interpretation session was attended by 9 participants who had completed both brainstorming and sorting activities. Four of them were service users and 5 of them were service providers. All the participants in the sorting and map interpretation round also identified as South Asian, regardless of if they were service users or providers (e.g. healthcare, community).

Tables 1 and 2 details the demographics we collected in the brainstorming, sorting and map interpretation activities. Almost all the participants identified as South Asian and female, with the majority of them being service users and having had at least one Pap test before. There was a wide range in the years that service providers had been engaged in their work, however the sorting and map interpretation round did not include people with less than 6 years of experience in their area of work. The majority of participants were between the ages of 31 years and 60 years, indicating that age-wise, most participants had not just become eligible, nor were they close to being ineligible, for cervical screening. During the sorting round, service providers indicated that between 9 and 85 per cent of the population they serve, is South Asian. During these activities, we also had representation from many different roles in health care and community services.

**Table 1:** Participant demographics asked during the brainstorming, sorting and map interpretation activities

Participant question	Options	Brainstorming (*n*=72)	Sorting (*n*=22)	Map Interpretation (*n*=9)
What best describes your role in this study?	I identify as South Asian	52	11	4
	I work in a role or an organization that serves South Asian women AND I identify as South Asian	13	8	4
	I work in a role or an organization that serves South Asian women AND I DO NOT identify as South Asian	1	0	0
	I work as a primary care provider AND I identify as South Asian	2	3	1
	I work as a primary care provider AND I DO NOT identify as South Asian	0	0	0
	Other	4	0	0
If you work in healthcare or in the community, how long have you been in this area of work?	1 to 5 years	5	0	0
	6 to 10 years	3	4	3
	11 to 15 years	4	1	1
	16 to 20 years	2	2	0
	20+ years	4	3	1
Have you ever had a Pap test?	Yes	46	13	9
	No	5	1	0
	Unsure	1	0	0
What is your age?	21 to 30	8	1	1
	31 to 40	19	6	2
	41 to 50	24	11	5
	51 to 60	13	4	1
	61 to 70	8	0	0
Do you identify as	Female	71	22	9
	Male	1	0	0
	Other	0	0	0

**Table 2:** Additional participant demographics asked during the sorting and map interpretation activities

Participant question	Options	Sorting (*n*=22)	Map Interpretation (*n*=9)
If you work in healthcare or in the community, what percentage of the population that you serve are South Asian?		9% to 85%	9% to 85%
If you work in healthcare or in the community, which of the following describes your role/work?^a^ (check all that apply)	Allied health professional (e.g. nurse, physiotherapist, dietician)	2	1
	Cancer care (screening, diagnosis, treatment)	2	2
	Community Outreach	2	1
	Health promoter	3	2
	Healthcare provider working in a hospital (e.g. hospitalist, inpatient nurse, mammography technician)	2	1
	Primary care provider	3	1
	Program coordinator	1	0
	Researcher	3	2
	Settlement services	2	1
	Volunteer	2	1

^a^only options that were chosen, are displayed here

We were successful in recruiting many South Asian women from a range of ages. While we had also strived to recruit service providers and were successful in recruiting many who worked in roles and organizations that serve South Asian women, we fell short in recruiting a large number of primary care providers such as family physicians and nurses who primarily do cervical screening in Ontario. Additionally, we only recruited 5 people who had never had a Pap test before.

### Experiences in the lives of South Asian women that shape their decisions around cervical screening: results from the brainstorming activity

Participants brainstormed a total of 210 statements and after idea synthesis, 45 unique and distinct statements were identified, to create the conceptual domain. These statements are listed in Table 3 and have been each assigned a number that is referenced throughout the results section and figures.

**Table 3:** Master list statements from the brainstorming round. Numbers are only to identify the statements throughout the text and figures. Numbers do not indicate rank value or any other value

Statement ID #	Statement
1	The belief that you should not “touch” things or go under the knife (meaning any medical procedure) because it brings more harm than good
2	Cultural expectations or pressures that the idea of “modesty” prevents women in the South Asian community from getting screened for cervical cancer.
3	Women do not go to the doctor unless they are having an issue
4	Appointments are not available at times that are convenient for patients
5	Women do not feel comfortable with their healthcare provider
6	Lack of access to cervical cancer screening information shared by trusted sources
7	Pap test appointments are viewed as time consuming
8	Women believing that a Pap test can lead to an infection
9	A woman’s lack of understanding and education around cervical cancer
10	Needing to communicate with healthcare providers in English is a barrier for South Asian women to be screened for cervical cancer
11	If a woman believes that cervical cancer is not a severe condition, this can discourage them from getting screened
12	Men in South Asian households make decisions about females getting screened
13	Education about cervical cancer is needed for men in South Asian households
14	A woman’s belief that cervical cancer screening is not necessary if you have only had one sexual partner
15	Women need reminders to know when they are due for cervical cancer screening
16	Negative cultural beliefs behind gynecologist visits leads to South Asian women feeling shame when booking appointments.
17	South Asian women are not comfortable to discuss their sexual history
18	Not enough media coverage of cervical cancer screening within the South Asian community
19	Pap tests can feel painful
20	Women may view a Pap test as a dirty procedure where you may bleed afterwards
21	Preventative care is not well understood by South Asian women
22	Prior negative experience with a Pap test discourages South Asian women from getting screened
23	South Asian women may be worried about their family finding out they are sexually active
24	Not having a healthcare provider of a similar cultural background makes intimate tests such as a Pap test, uncomfortable
25	Sex is a taboo topic amongst South Asians
26	Any tests related to sex can be considered dirty
27	Women believe that if they have an HPV vaccine, they do not need to be screened for cervical cancer
28	Women may be shy to have an examination in that area of their body
29	Foreign trained physicians may not encourage their patients to do cancer screening, as preventative care may not have been common in their home countries.
30	South Asian women may prioritize looking after their families over their own health
31	South Asian women may be too busy with their jobs or careers to take care of their own health
32	Lack of support from family members to go and get screened
33	Lack of support from friends to go and get screened
34	Women are afraid to find out if they have cancer
35	Cervical cancer screening is not openly discussed in the South Asian culture
36	Women may be uncomfortable with going to the doctor in general
37	Women hear other women share negative experiences about getting a Pap test
38	The belief that if a cervical cancer diagnosis is your fate or destiny, there is no reason to get screened
39	Belief that you only have to worry about cervical cancer if you have a problem with your menstruation
40	Family doctor does not encourage cervical cancer screening during appointment
41	Women may not know what a Pap test involves
42	Women may not know the purpose of a Pap test
43	Women do not have a family doctor
44	South Asian women will only get screened when symptoms arise
45	South Asian women won’t get screened because they think they cannot get cervical cancer.

Statements about South Asian culture came up often during the brainstorming. Participants talked about how it can lead to discomfort during healthcare visits related to women’s health: ‘negative cultural beliefs behind gynecologist visits leads to South Asian women feeling shame when booking appointments’ (statement #16); and ‘cultural expectations or pressures that the idea of “modesty” prevents women in the South Asian community from getting screened for cervical cancer’ (#2). What was particularly common amongst the statements were references to sexual intercourse. Statements such as #25 and #26 show how discomfort around the topic of sexual intercourse can impact how people view and participate in cervical screening, as being sexually active is an eligibility criterion.

The brainstorming activity also uncovered a range of personal beliefs. Fatalistic attitudes (#38) show that some women may not get screened because they think if it is their destiny to get cancer, screening or any other preventive measure is pointless. Statements also reflected how fear and uncertainty can stand as a barrier to screening (#1 and #34). Some misconceptions that may convince South Asian women that they do not need cervical screening came up (#14, #27, and #45). Additionally, statement #11 demonstrates how some women may not feel the need to get screened, if they do not view cervical cancer as particularly threatening to their well-being.

Statements about the Pap test itself, were largely present in the brainstormed list. A lack of understanding around Pap tests is highlighted in statements such as #41 and #42. The statements also demonstrated that the Pap test itself can be a barrier to continued participation in cervical screening as it can be viewed as painful (#19), possibly leading to an infection (#8), and time consuming (#7). Having or hearing about prior negative experiences with Pap tests can also impact future screening participation as shown in the statements #22, #28 and #37.

### Sorting and creation of cluster map to understand how participants thought experiences relate to each other and cluster into larger themes

The statements in the master list were then sorted by participants and this sorting data was used to populate a map based on how often the statements were sorted together and create the basis for the conceptual framework. Of the 22 participants that completed sorting, data from 18 were included in the analysis. Four participants’ sorting data was not used because of errors in their sorting (e.g. too few statements were sorted, statements were sorted multiple times).

**Fig. 2:**
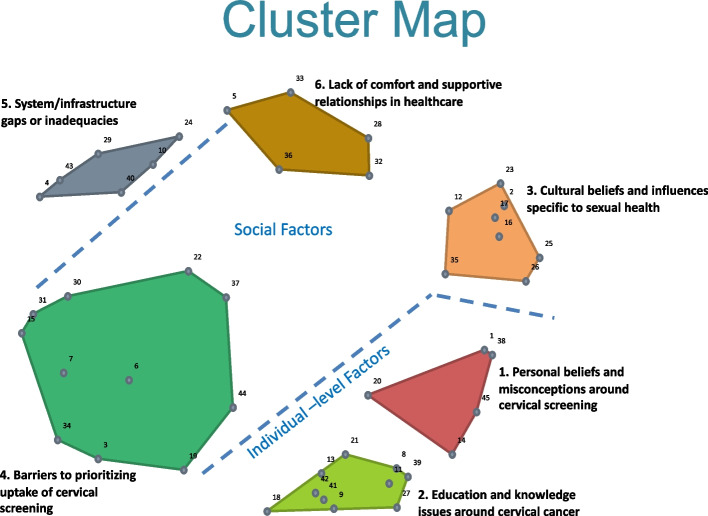
Cluster map labelled with themes and regions. Numbers are only to identify the statements and clusters throughout the text and figures. Numbers do not indicate rank value or any other value

Figure 2 presents the cluster map with the location of each statement, represented as a numbered point that corresponds to Table 3. The stress value is 0.2780 after 16 iterations, suggesting a good overall fit between the sorted data and the produced map.

During the map interpretation session, participants agreed on the 6-cluster solution for the map as they agreed with the ways in which statements were grouped together and the distinctions that were made by these grouping. There was some discussion about moving individual statements from one cluster to another, but overall there was no complete agreement amongst the participants for moving any individual statement from one cluster to another. As a result, no statements were moved.

The final cluster labels determined during the map interpretation session were: 1) Personal beliefs and misconceptions around cervical cancer; 2) Education and knowledge issues around cervical cancer; 3) Cultural beliefs and influences specific to sexual health; 4) Barriers to prioritizing uptake of cervical screening; 5) System/ infrastructure gaps or inadequacies; and 6) Lack of comfort and supportive relationships in healthcare. During the session, two distinct map regions were also identified. Cluster 1 and 2 in the bottom right corner of the map, were labelled as a part of the map that is mainly representative of ‘individual-level factors.’ Clusters 3, 4, and 6 were seen as the area of the map that represented ‘social factors.’ Cluster 5 was believed to be distinct from the two regions as it was specific to healthcare spaces and delivery. This showed us, thematically, what about the lives and experiences of South Asian women impact their decisions to get screened for cervical cancer.

#### Individual level factors region

Cluster 1 ‘personal beliefs and misconceptions around cervical screening’ contained statements representing fatalistic attitudes (#38,#1) and misconceptions that convince people they do not need to get screened (#14), including the belief that if they only ever had one sexual partner they do not need to be screened, or simply that they cannot get cervical cancer (#45). In this same region was cluster 2, ‘education and knowledge issues around cervical cancer’ which contained broad topics such as understanding preventative care, as well as more specific topics such as details about a Pap test, were covered. Overall, education around cervical cancer and related screening largely made up cluster. There were some topics that seem closely related to cluster 1 – a matter that was also brought up by two participants during map interpretation – and those were the statements that reflect misconceptions (#27 and #39). While these statements were not located close enough to be placed in Cluster 1 by GroupWisdom, it is important to note that cluster 1 and cluster 2 are located close to each other in the map, further supporting a demarcation of the ‘individual-level factors’ region in the bottom right corner of the map.

#### Social factors region

In cluster 3, ‘cultural beliefs and influences specific to sexual health’ the statements reflected the impact of South Asian culture on sexual health. Statements such as #25 and #17 demonstrate some of the social barriers that exist for women to openly discuss their sexual health. The statements in this cluster also show how these cultural beliefs can then impact perceptions of Pap tests. This region also contains cluster 4, ‘barriers to prioritizing uptake of cervical screening’ this is the largest cluster on the map. Here, statements around aspects of people lives and experiences that impact how and if they prioritize screening, were largely represented. Statements #30 and #31 demonstrate how aspects of people’s day-to-day lives can impact health actions such as cervical screening. Overall approaches to health and healthcare such #3 and #44 demonstrate how cervical screening may go unprioritized because people are asymptomatic, as well as experiences with the Pap test itself seemed to be a driver for people to avoid cervical screening. Cluster 6, ‘lack of comfort and supportive relationships in healthcare’ contains statements around the impact of family and friends as sources of support to get screened (#32 and #33). Other statements in this cluster showed the role of discomfort with healthcare providers (#5 and #36)– and having an intimate examination (#28). These statements suggest that support from friends and family, as well as comfort in the screening procedure, impact screening.

#### System/infrastructure gaps or inadequacies

Cluster 5 was seen as distinct from the other clusters and regions by participants in the map interpretation session. This cluster contains statements related to patient-provider interaction (#29, #40), provider characteristics (#24), language needs (#10), availability of providers (#4) and overall access to healthcare (#43).

### Bridging values

Table 4 presents the average bridging value for each cluster. Cluster 2 (education and knowledge issues around cervical cancer) has an average bridging value close to 0, indicating that this cluster is conceptually clear from the rest of the map, and that the statements in this cluster were very often sorted together by participants. The low bridging values indicate that this is a distinct area of the map with little to no relationships with other areas of the map. This supports the decision to not combine Clusters 1 and 2 together – as proposed in the 5 cluster solution - and to keep them distinct. This tells us that education and knowledge around cervical cancer are viewed as distinct factors, that play a role in cervical cancer screening.

**Table 4:** Bridging values for each cluster. Cluster ID numbers are only to identify the clusters throughout the text and figures. Cluster ID numbers do not indicate rank value or any other value

Cluster ID #	Cluster Name	Bridging
1	Personal beliefs and misconceptions around cervical screening	Avg 0.27
2	Education and knowledge issues around cervical cancer	Avg 0.08
3	Cultural beliefs and influences specific to sexual health	Avg 0.28
4	Barriers to prioritizing uptake of cervical screening	Avg 0.61
5	System/infrastructure gaps or inadequacies	Avg 0.27
6	Lack of comfort and supportive relationships in healthcare	Avg 0.48

Clusters 1 and 3 also have relatively low average bridging values, further indicating this area of the map is conceptually clear (i.e. participants did not vary in how they viewed them as related to other statements in this area of the map) with statements in this area often being sorted with adjacent statements and less likely with those across the map. This is also true for Cluster 5 which is located on the other side of the map and has an average bridging value of 0.27. This further supports the participants in the map interpretation session who thought Cluster 5 was distinct from the rest of the clusters and regions and was therefore a region of its own. Overall, these values also tell us that there is greater cohesion amongst the statements within the three clusters, and low variability amongst participants’ sorting. These statements are closely related to each other, compared to areas of the map with higher values, meaning that the ideas in these clusters are not related closely to other areas of the map.

Parts of the map with higher bridging values indicate statements that are ‘bridges’ between areas of the map. Cluster 4 has a relatively higher average bridging value of 0.61indicating a higher variability in how participants interpreted and sorted the statements within the cluster. This does not mean that these statements were not as conceptually clear, but rather that participants varied in how they saw them as related to other statements on the map.

Examining areas of the map with many anchored statement areas where participants were consistently grouping items together, we can see that statements in the bottom right corner of the map (Fig. 2) have very low bridging values. Statements #8, #11 and #27 have very low bridging values. This indicates that participants consistently sorted these statements together, and this area of the map is conceptually clear and distinct from other ideas throughout the map. These statements are anchors. This supports the decision to not move these individual statements out of cluster 2, as mentioned earlier.

## Discussion

This concept mapping analysis identified how individual-level factors around personal beliefs and misconceptions, as well as education and knowledge issues around cervical cancer, impact screening amongst South Asian women. We found that larger, social impacts around themes of cultural beliefs and influences specific to sexual health, barriers to prioritizing uptake of cervical screening, as well as lack of comfort and supportive relationships in healthcare, can also play a role. Lastly, we found that despite these social and individual-level factors, issues within the healthcare system can be a determinant around access to screening.

### Implications for policy and health intervention

Currently the province of Ontario is still working to recover from screening shutdowns and widening of screening gaps during the COVID-19 pandemic. The backlog of cancer screening and services in the province is around 1.1 million from the first year of the COVID-19 pandemic alone [41]. Furthermore, with increasing evidence around the efficacy of HPV screening, Ontario will be moving towards HPV testing in the coming years [6] with some parts of Canada already making the switch [42, 43], The World Health Organization has set out with the goal of eliminating cervical cancer by 2040 through screening with a high performance test equal to, or better than, an HPV test [44]. HPV testing is critical to cervical cancer prevention, and implementing routine HPV testing can put all countries on the path to eliminating cervical cancer [44]. In line with the WHO goals, the Canadian Partnership Against Cancer (CPAC) currently has a goal of elimination of cervical cancer in Canada by 2040 through HPV immunization and screening [45]. The findings from this concept mapping analysis highlight critical points of intervention where current rates of underscreening can be addressed, while also identifying important considerations for implementing HPV screening. For example, discomfort with the Pap test – physical pain, shyness, concerns around bleeding and infections – was highlighted by many statements from the brainstorming round. Studies have shown the acceptability of HPV self-sampling [22, 23] – a test that is less physically invasive and can be self-administered when and wherever someone chooses – for cervical screening amongst under or never screened (UNS) people. This CM analysis further underscores how promising HPV self-sampling can be for groups of people that are largely under screened. As the province of Ontario designs their implementation of HPV testing for cervical screening, it would be important to include self-collection alongside physician-collection HPV tests for UNS people.

The role of culture was present in many ways, including how and if sex is openly discussed, as well as gender roles and decision-making within South Asian households. While challenging, addressing sex being a taboo topic, and educating male partners and other household members about cervical cancer, will be an important area to address in the coming years. Additionally, the role of comfort and supportive relationships could see the increased implementation of peer support programs [46] that has already been shown as promising in cervical screening. This can also address the many statements around education and knowledge issues, as well as misconceptions that exist within the South Asian and other under screened communities.

### Comparison with other studies

Other studies done in this area, similarly identified the role of fatalistic attitudes in cervical screening. Literature has shown that some South Asian women have personal convictions that getting cancer is predestined or karma for past actions, so there is little belief that screening can make a difference [20, 28, 47, 48]. Misconceptions such as the purpose of Pap tests being for sexually transmitted infections [19] or that healthy lifestyles prevent cancer [5] can also cause women to choose not to be screened.

Literature shows that physicians can be seen as authoritative and trustworthy, and if they do not recommend screening, women may not think it is important [10], and this was similarly seen in the concept map where patient-provider interactions was found to be a distinct area. Past experiences, including feeling rushed and unheard and painful examinations can discourage screening [24, 49], which can further explain the statement around how some women may generally be uncomfortable going to the doctor. We also found that the social identity of healthcare providers matter. Other studies show that the gender of health care providers, can play a role, as many South Asian women have shown a preference for a female provider to perform examinations and Pap tests, and asking for a referral to a female provider from a male provider is not always successful [17, 28, 50]. Lastly, literature further elaborates around the stigma within certain South Asian communities around sexual activity, as a woman getting screened would imply certain details about their sexual activity including the virginity of unmarried women [17, 18, 24, 28].

While literature shows that family and friends can influence the uptake of screening by validating concerns, sharing experiences and providing advice, they can, in some cases also be discouraging or forbid screening [20, 24, 49]. Our analysis further elaborated on this by uncovering the role of men and other household members in the decision to get screened, and how education around cervical screening is also needed for them.

### Limitations

The main limitation of our study is that conversational English was required of participants. Since concept mapping largely involves participants brainstorming and then interpreting statements, it would be challenging to accommodate multiple languages, even with the assistance of interpreters and translation, as the meanings of individual statements may be lost in between translations. Language poses a barrier for some South Asian women to receive healthcare, and therefore this study may exclude a subset of women who are UNS for cervical cancer. Additionally, our small subgroup of primary care providers may have meant we were unable to uncover even more experiences from clinical encounters (e.g. a Pap test).

Furthermore, while this study rationale assumes there are experiences and realities of South Asian women that are different from the larger population and therefore should be studied to understand underscreening in the South Asian community, it is also critical to understand the diversity amongst women who identify as South Asian. Amongst South Asian women, there is much diversity along such lines as ethnicity, religion, age, social class, sexual orientation, education and marital status.

Lastly, while the anonymous weblink used in the brainstorming round had its benefits to encourage more participants to discuss an often-uncomfortable topic, it also meant we were unable to report on an exact number of individuals who participated in that activity. Further our use of a zoom link for the map interpretation made geography and transportation less of a barrier for participation, however in using this platform we may have excluded participants without access to a private setting and/or videoconferencing equipment (e.g. camera, microphone).

## Conclusions

With the participant-driven method of concept mapping, we were able to uncover a range of factors in the lives and experiences of South Asian women that impact decisions around cervical screening, and the interrelationships amongst these factors. From this we were able to develop a conceptual framework to understand how the lives and experiences of South Asian women shape their decisions around getting screened for cervical cancer. While previous studies may have uncovered ideas similar to the 45 statements, this work goes a step further to show how people relate these individual ideas to each other and to the larger themes, within and outside healthcare. We uncovered particular issues with Pap tests, relationships impacting healthcare (providers, friends and family), personal beliefs, as well as knowledge issues around cervical cancer and preventative care. We identified specific cultural and social factors amongst South Asian communities that impact cervical screening, including sex being a taboo topic and gender roles that impact prioritization and decision-making. We also uncovered the larger interrelationships between statements considered to be barriers to prioritizing screening and other ideas throughout the map. The participants thematically organized the ideas into 6 different clusters, but overall saw delineation along the lines of individual, social and healthcare system level factors that impact cervical screening participation.

The findings demonstrate that multiple interventions that cross-cut multiple levels are needed, as culture, society, healthcare, and other larger structures influence individual actions. This can include interventions that address both individual and cultural understandings of cervical screening and cancer, as well as women’s health. Personal beliefs and misconceptions appear to play a role in South Asian women’s screening actions, and as such addressing them through both individual-level and community-level interventions will be important. Additionally, support is needed for women both at the community and system-levels, as this came up as major thematic groupings in the sorting data. Engaging community to further understand what this entails is needed. Next steps in this work are to further analyse the data to understand priorities for action amongst the generated statements.

## Data Availability

The datasets generated and/or analyzed during the current study are not publicly available due to maintaining the privacy and confidentiality of participants, but are available from the corresponding author on reasonable request.
